# A longitudinal, observational study of the features of transitional healthcare associated with better outcomes for young people with long-term conditions

**DOI:** 10.1186/s12916-018-1102-y

**Published:** 2018-07-23

**Authors:** A. Colver, H. McConachie, A. Le Couteur, G. Dovey-Pearce, K. D. Mann, J. E. McDonagh, M. S. Pearce, L. Vale, H. Merrick, J. R. Parr, On behalf of the Transition Collaborative Group, Caroline Bennett, Greg Maniatopoulos, Tim Rapley, Debbie Reape, Nichola Chater, Helena Gleeson, Amanda Billson, Anastasia Bem, Stuart Bennett, Stephen Bruce, Tim Cheetham, Diana Howlett, Zilla Huma, Mark Linden, Maria Lohan, Cara Maiden, Melanie Meek, Jenny Milne, Julie Owens, Jackie Parkes, Fiona Regan, Nandu Thalange

**Affiliations:** 1 Institute of Health & Society, Sir James Spence Institute, Royal Victoria Infirmary, Newcastle University, Queen Victoria Road, Newcastle upon Tyne, NE1 4LP UK; 2 Northumbria Healthcare NHS Foundation Trust, North Tyneside General Hospital, Rake Lane, North Shields, NE29 8NH UK; 3 Northumberland, Tyne and Wear NHS Foundation Trust, St. Nicholas Hospital, Jubilee Road, Newcastle upon Tyne, NE3 3XT UK; 4 Centre for Musculoskeletal Research and Manchester Academic Health Science Centre, University of Manchester, Stopford Building, Oxford Rd, Manchester, M13 9PT UK; 5 NIHR Manchester Biomedical Research Centre, Manchester University NHS Foundation Trust, Manchester Royal Infirmary, Oxford Rd, Manchester, M13 9WL UK; 6 Institute of Neuroscience, Sir James Spence Institute, Newcastle University, Queen Victoria Road, Newcastle upon Tyne, NE1 4LP UK; 7 Great North Children’s Hospital, Newcastle Upon Tyne Hospitals NHS Foundation Trust, Royal Victoria Infirmary, Queen Victoria Road, Newcastle upon Tyne, NE1 4LP UK

**Keywords:** Transition, Adolescence, Health service delivery

## Abstract

**Background:**

Most evidence about what works in transitional care comes from small studies in single clinical specialties. We tested the hypothesis that exposures to nine recommended features of transitional healthcare were associated with better outcomes for young people with long-term conditions during transition from child-centred to adult-oriented health services.

**Methods:**

This is a longitudinal, observational cohort study in UK secondary care including 374 young people, aged 14–18.9 years at recruitment, with type 1 diabetes (*n* = 150), cerebral palsy (*n* = 106) or autism spectrum disorder with an associated mental health problem (*n* = 118). All were pre-transfer and without significant learning disability. We approached all young people attending five paediatric diabetes centres, all young people with autism spectrum disorder attending four mental health centres, and randomly selected young people from two population-based cerebral palsy registers. Participants received four home research visits, 1 year apart and 274 participants (73%) completed follow-up.

Outcome measures were Warwick Edinburgh Mental Wellbeing Scale, Mind the Gap Scale (satisfaction with services), Rotterdam Transition Profile (Participation) and Autonomy in Appointments.

**Results:**

Exposure to recommended features was 61% for ‘coordinated team’, 53% for ‘age-banded clinic’, 48% for ‘holistic life-skills training’, 42% for ‘promotion of health self-efficacy’, 40% for ‘meeting the adult team before transfer’, 34% for ‘appropriate parent involvement’ and less than 30% for ‘written transition plan’, ‘key worker’ and ‘transition manager for clinical team’.

Three features were strongly associated with improved outcomes. (1) ‘Appropriate parent involvement’, example association with Wellbeing (b = 4.5, 95% CI 2.0–7.0, *p* = 0.001); (2) ‘Promotion of health self-efficacy’, example association with Satisfaction with Services (b = − 0.5, 95% CI – 0.9 to – 0.2, *p* = 0.006); (3) ‘Meeting the adult team before transfer’, example associations with Participation (arranging services and aids) (odds ratio 5.2, 95% CI 2.1–12.8, *p* < 0.001) and with Autonomy in Appointments (average 1.7 points higher, 95% CI 0.8–2.6, *p* < 0.001).

There was slightly less recruitment of participants from areas with greater socioeconomic deprivation, though not with respect to family composition.

**Conclusions:**

Three features of transitional care were associated with improved outcomes. Results are likely to be generalisable because participants had three very different conditions, attending services at many UK sites. Results are relevant for clinicians as well as for commissioners and managers of health services. The challenge of introducing these three features across child and adult healthcare services, and the effects of doing so, should be assessed.

**Electronic supplementary material:**

The online version of this article (10.1186/s12916-018-1102-y) contains supplementary material, which is available to authorized users.

## Background

Young people with long-term conditions have a physical, mental or health impairment with the potential for a substantial and long-term adverse effect on their everyday lives [1]. Adolescence and young adulthood is a key developmental stage, extending into the mid-twenties, during which a young person experiences many developmental transitions such as leaving school, gaining training, employment or further education, forming romantic relationships and potentially leaving home. Simultaneously, the healthcare of young people with a long-term health condition ‘transfers’ from child to adult health services, with the expectation that young people take increasing responsibility for managing their health condition. Many young people with long-term health conditions have poor health and social outcomes following transition [2, 3]. The importance of healthcare transition and its challenges are recognised in the 2016 UK National Institute for Health and Clinical Excellence (NICE) Guideline 43 [4] and Quality Standard 140 [5]. ‘Transition’ is the purposeful, planned process that addresses the medical, psychosocial and educational/vocational needs of adolescents and young adults with long-term conditions as they move from child-centred to adult-oriented healthcare systems [6]. ‘Transfer’ is the formal event when the healthcare of a young person moves from children’s services to adults’ services.

The international research literature proposes service features that might promote better healthcare transition, both at national level [7, 8] and specialty level [9]. However, there is a lack of evidence about whether these ‘proposed beneficial features’ improve outcomes [10]. A systematic review [11] highlighted some evidence, mainly from diabetic services, and concluded that the most encouraging interventions were those oriented to patients (educational programmes and skills training), staffing (transition co-ordinators), and service delivery (young adult clinics or enhanced follow-up). The recent evidence overview, provided by NICE Guideline 43 [4], set out 9 overarching principles and 47 recommendations but recognised it could cite relatively little high-quality evidence to support them or to prioritise them.

Recommendations for particular service features should be supported by robust evidence that indicate improved outcomes across a range of conditions and settings before they are adopted into practice. With this challenge in mind, we designed our research to enable us to examine patient-level outcomes that would be applicable across a range of conditions. We focused on nine proposed beneficial features (PBFs, see Methods). The aim of this longitudinal, observational study was to test the hypothesis that exposure to these PBFs is associated with better outcomes for young people with long-term conditions, namely satisfaction with services, mental wellbeing, participation and autonomy in appointments.

## Methods

The study methods and sample characteristics, described in detail elsewhere [12, 13], are summarised below.

### Participants

The study recruited 374 young people from across England and Northern Ireland, on the basis of having one of three conditions – 150 young people with type 1 diabetes mellitus (exemplar of chronic health condition with national standards of care, recruited through five NHS Trusts); 118 young people with autism spectrum disorder (ASD) and additional mental health problems (exemplar of neurodevelopmental disorder, recruited through four NHS Trusts); and 106 young people with cerebral palsy (CP) (exemplar of complex physical disability, recruited through two regional population registers and one NHS Trust). Young people were aged 14 to 18.9 years at recruitment, did not have significant learning disability, and had not transferred to adult healthcare. For each young person, a parent or carer was also invited to participate.

### Procedure

Recruitment was between June 2012 and October 2013. Local researchers visited the young people and parents, usually at home, took informed consent and administered independently completed questionnaires. Visits were arranged annually for 3 years. The local researchers attended joint training each year, and participated in group telephone discussions at around 3-month intervals to maintain consistency of approach. To maximise young person engagement and retention, outcome measures could be completed by post or electronically. At the baseline visit, the nature of the PBFs was discussed with the young person and a log-book was provided. Before each subsequent annual visit, the researcher consulted the young person’s medical records to seek evidence of the PBFs having been provided (for example, inclusion of a member of the adult service at a paediatric appointment). Then, at the visit, the researcher and young person completed a summary sheet recording whether each PBF had been experienced or not in the previous year; the information gathered from medical notes acting as additional prompts for the discussion.

### PBFs

Definition of the nine PBFs is provided in Box 1. ‘Appropriate parent involvement’ represents the perceptions of both the young person and parent being satisfied with level of parent involvement. These were chosen on the basis of being recommended in recent guidance [4], identified as beneficial in a systematic review [11, 14], and following our own analysis of individual studies (Table two in Colver et al. [12]).

### Outcome measures

We chose measures of satisfaction with services, mental wellbeing, participation and autonomy in appointments. They were chosen to be relevant across conditions and settings and correspond to measures subsequently proposed in international Delphi studies [15, 16]. In particular, we included a measure of mental wellbeing which captures what the young person feels about their life, as recommended by an International, Interdisciplinary Health Care Transition Research Consortium [15, 17]. The measure of participation reflects the importance that the International Classification of Functioning, Disability and Health [18] attaches to social as well as health outcomes.

Satisfaction with services was assessed using Mind the Gap [19]. This scale measures the difference or ‘gap’ between a young person’s ideal service and the service they have received (thus higher scores indicate lower satisfaction). Service satisfaction is expressed as a total score, with subdomain scores for Management of the environment, Provider characteristics and Process issues.

Mental wellbeing was assessed using the Warwick Edinburgh Mental Wellbeing Scale [20]. This 14-item questionnaire collects responses to each item on a 5-point Likert scale (‘none of the time’ 1 to ‘all of the time’ 5). Higher scores (range 14–70) denote higher mental wellbeing.

Participation was measured by the Rotterdam Transition Profile [21]. This captures independence in participation across nine domains. Independence is categorised into three ‘phases’, phase 1 being the least independent (thus higher scores indicate higher participation). A further participation measure, not in the original protocol, was added on the advice of the Programme’s External Advisory Board. This was Autonomy in Appointments, involving three questions about whether the young person makes their own appointments and asks and answers questions themselves (range 3–15) [22]. Higher scores indicate greater autonomy.

### Other information

Baseline demographic information, including age, sex, index of multiple deprivation (IMD) [23] calculated from postcode in England, and the multiple deprivation measure [24] in Northern Ireland, and a number of socioeconomic indicators, including family composition, was collected for those who participated in the study.

‘Date of transfer’ was defined as the date of the last appointment with a paediatrician or adolescent psychiatrist. The young person’s status at each visit was recorded as still in a children’s service, transferred to dedicated adult service, transferred to General Practitioner in primary care, or lost to follow-up.

In order to maximise useable questionnaire data, the ‘final visit’ was defined as visit 4, or as visit 3 if visit 4 did not take place or questionnaire data were missing.

Our protocol [12] also proposed condition-specific outcomes and data relevant to economic analysis; these data will be or have been reported elsewhere [25, 26].

### Data analysis and statistical methods

Age, Satisfaction with services (total and per domain), Mental wellbeing, and Autonomy in appointments were treated as continuous variables. Condition, site, sex, participation and exposure to PBFs were treated as categorical variables. Missing data were handled according to the suggested rules for each outcome measure. In the regression analyses, those with missing data for PBFs (year-by-year and consolidated PBF indicator) were grouped together and included in the modelling.

Representativeness between young people who were retained to final visit and those lost to follow-up, by age, sex, condition, site and socioeconomic status, was assessed using t, Mann–Whitney and χ^2^ tests as appropriate. The Kruskal–Wallis test was used to examine associations between outcome measures and condition. Comparisons of outcome measures between baseline and final visit were tested using Wilcoxon paired sign-rank and χ^2^ tests.

Two approaches to analysis were undertaken to assess the association of PBFs with outcomes across the duration of follow-up. The first approach used the young person’s experience of each PBF during the previous year (i.e. whether the PBF was present (yes) or absent (no) during that year) and tested this against each outcome at the end of that year. Hence, analyses were conducted ‘year-by-year’ and each was a cross-sectional analysis. The time between baseline (visit 1) and visit 2 was called ‘Period’ 1; similarly for periods 2 and 3.

The second approach used the young person’s experience of each PBF throughout the 3 years of the study. This was a longitudinal analysis. It was not appropriate to model the exposure to PBFs by simple frequencies, as young people had varying numbers of contacts with clinicians; in some years, a young person experienced a PBF and the next year might not. Also there was variation in the times at which the PBFs were captured; although intended to be every year, appointments often fell 1 or 2 months either side for practical reasons. We therefore developed a consolidated indicator to estimate the extent to which each PBF was delivered over the duration of the study. The indicator was based on whether the PBF had been experienced or not in each follow-up period. It was defined to be ‘optimal’ or ‘sub-optimal’ for each PBF, based on the following criteria developed by consensus within the members of the research team:

- Group one optimal: for ‘age-banded clinic’, ‘meet adult team before transfer’, ‘written transition plan’, ‘holistic life-skills training’ and ‘transition manager for clinical team’; evidence that the PBF was experienced or recorded in the 12 months before at least one of the research visits 2, 3 and 4 over the 3 years.
- Group two optimal: for ‘key worker’ and ‘coordinated team’; the PBF should have been experienced in the 12 months before at least two of the research visits 2, 3 and 4 over the 3 years.
- Group three optimal: for ‘promotion of health self-efficacy’ and ‘appropriate parent involvement’; the PBF should have been experienced in the 12 months before all research visits 2, 3 and 4 over the 3 years.

For both approaches, linear or logistic regression modelling was used, depending on the nature of the outcome variable, to test for association between each PBF individually or each consolidated PBF indicator and outcomes. All models were adjusted for age, sex, condition and potential for clustering by site.

Significant associations (*p* < 0.05) from these models were further adjusted for transfer status, time since transfer to final visit (if applicable) and time to first adult appointment (if applicable).

A significance threshold of *p* ≤ 0.01 was used in final models to mitigate multiple testing (but associations at *p* ≤ 0.05 are presented as supporting evidence). Data were analysed using STATA version 14.

### Patient involvement

Throughout the 5 years of the Transition Research Programme, a young persons’ advisory group (UP) met each month. All group members had long-term health conditions. The group provided advice on outcome measures, recruitment and data collection, and interpretation of findings. Details of UP’s activities are on the Programme’s website: http://research.ncl.ac.uk/transition/.

To support recruitment and retention during the study, all participants and referring clinicians received regular newsletters (approximately every 9 months) about the progress of the study. Feedback of the results at the end of the study to participants took place in two ways, (1) through displaying results of the research on the website, and (2) a summary of the results was included in the final newsletter sent to every participant who wanted to continue to receive information about the study.

## Results

A total of 374 young people were recruited to the study (150 for diabetes, 106 for CP, 118 for ASD), mean age 16.2 years (standard deviation (SD) 1.3), along with 369 parents/carers. Demographic data are summarised in Additional file 1: Table S1. As previously reported [13], participants did not differ significantly from non-participants by age or sex. Overall, participants had significantly (*p* < 0.001) lower socioeconomic status scores (i.e. less deprived) than non-participants; however, the difference in overall IMD score on a continuous scale ranging from 0.5 to 87.8, was only 6.1. Further, the proportion of single parent families with dependent children in the UK Annual Families and Households Survey 2013 [27] was 25.1%, very similar to the proportion in our sample at 23.7% (Additional file 1: Table S1).

### Attrition

A total of 304 (81.3%) young people remained in the study by visit 2, 259 (69.3%) by visit 3 and 274 (73%) by final visit (235 from visit 4 and 39 from visit 3, see Methods). Of these 274 young people, 58% were male and there were 112 with diabetes, 74 with CP and 88 with ASD. The mean time between baseline visit and final visit was 2.9 years (SD 0.4, range 1.8–3.9).

There were no significant differences between those remaining in the study and those not remaining with respect to sex (*p* = 0.6), age (*p* = 0.6), condition (*p* = 0.6), diabetes sites (*p* = 0.4) or ASD sites (*p* = 0.6). However, in Northern Ireland, those with CP lost to follow-up came from areas with, on average, greater socioeconomic deprivation (*p* = 0.03). Examining socioeconomic factors based on actual circumstances rather than area of residence, there was a significant reduction in the proportion of families with single parents (Additional file 1: Table S1).

Of the 100 participants not remaining in the study, one had died, 28 were lost to follow-up and 71 withdrew. Of those withdrawing, 22 said they were no longer interested, 19 had other commitments, 19 experienced personal issues such as major injuries or severe parental illness, and the remainder gave miscellaneous reasons.

The mean age at final visit was 19.1 years (SD 1.4, range 16.1–22.0). Of the 274 participants at final visit, 49 (18%) remained in child services and 225 (82%) had left child services. Very different proportions by condition transferred to primary care (General Practice) as compared to a dedicated adult service (Table 1).

**Table 1:** Service attended by young people at final visit

Service	All	D	CP	ASD
	n (%)	n (%)	n (%)	n (%)
Remained in child services	49 (18)	19 (17)	10 (14)	20 (23)
Left child services:	225 (82)	93 (83)	64 (86)	68 (77)
To adult services	149	90	35	24
To primary care (General Practitioner)	76	3	29	44

*D* diabetes, *CP* cerebral palsy, *ASD* autism spectrum disorder

### Changes in outcomes during the study

The average changes in outcomes between baseline and final visits are shown in Additional file 1: Table S2. In summary, satisfaction with services decreased overall but remained stable for those with diabetes; mental wellbeing was steady overall but was always lower for those with ASD and associated mental health problems; participation increased overall but was always higher for those with diabetes; and autonomy in appointments increased overall but was again always higher for those with diabetes.

### PBFs experienced over transition

Table 2 sets out the extent to which participants experienced optimal or suboptimal exposure to PBFs across the period of the study, using the consolidated indicator. Optimal exposure to features of transitional healthcare was 61% for ‘coordinated team’, 53% for ‘age-banded clinic’, 48% for ‘holistic life-skills training’, 42% for ‘promotion of health self-efficacy’, 40% for ‘meeting the adult team before transfer’, 34% for ‘appropriate parent involvement’, and less than 30% for ‘written transition plan’, ‘key worker’ and ‘transition manager for clinical team’. Significantly more young people with diabetes experienced optimal exposure to PBFs compared to those with CP or ASD, particularly ‘meeting the adult team before transfer’ and ‘promotion of health self-efficacy’.

**Table 2:** Consolidated indicator of Proposed Beneficial Features at final visit, by condition group

Consolidated indicator of Proposed Beneficial Feature		n	%	n	%	n	%	n	%	*p* value
		All		D		CP		ASD		
Age-banded clinic	Optimal	145	53	109	97	16	22	20	23	< 0.001
	Sub-optimal	111	40	2	2	54	73	55	62	
	Missing	18	7	1	1	4	5	13	15	
Meet adult team before transfer	Optimal	111	40	73	65	16	22	22	25	< 0.001
	Sub-optimal	133	49	31	28	54	73	48	55	
	Missing	30	11	8	7	4	5	18	20	
Promotion of health self-efficacy	Optimal	116	42	76	68	18	24	22	25	< 0.001
	Sub-optimal	151	55	29	26	56	76	66	75	
	Missing	7	3	7	6	0	0	0	0	
Written transition plan	Optimal	48	17	32	29	11	15	5	6	< 0.001
	Sub-optimal	185	68	62	55	59	80	64	73	
	Missing	41	15	18	16	4	5	19	21	
Appropriate parent involvement										
Both young person and parent happy with parent involvement	Optimal	93	34	36	32	28	38	29	33	0.44
	Sub-optimal	141	51	55	49	33	45	53	60	
	Missing	38	14	21	19	12	16	5	6	
	Non-applicable	2	1	0	0	1	1	1	1	
Key worker	Optimal	79	29	56	50	3	4	20	23	< 0.001
	Sub-optimal	170	62	47	42	68	92	55	62	
	Missing	25	9	9	8	3	4	13	15	
Coordinated team	Optimal	167	61	104	93	25	34	38	43	< 0.001
	Sub-optimal	66	24	2	2	40	54	24	27	
	Missing	25	9	6	5	4	5	15	17	
	Non-applicable	16	6	0	0	5	7	11	13	
Holistic life-skills training	Optimal	132	48	74	66	18	24	40	45	< 0.001
	Sub-optimal	117	43	28	25	52	70	37	42	
	Missing	25	9	10	9	4	6	11	13	
Transition manager for clinical team	Optimal	60	22	27	24	14	19	19	21	0.95
	Sub-optimal	143	52	67	60	34	46	42	48	
	Missing	71	26	18	16	26	35	27	31	
Total n		274		112		74		88		

*D* diabetes, *CP* cerebral palsy, *ASD* autism spectrum disorder

### PBFs as predictors of Mind the Gap scores

In the year-by-year analysis, there were significant positive associations (*p* ≤ 0.01) during each period between ‘appropriate parental involvement’ and satisfaction with services (Mind the Gap) overall and in most domains of the instrument (Table 3). These were confirmed by significant associations of the consolidated PBF indicator with Mind the Gap at final visit.

**Table 3:** Associations of Proposed Beneficial Features (PBFs) with outcome measures

	PBFs by ‘year-by-year’ visits					Consolidated PBF indicator at final visit			
PBF	Period	Outcome	Coefficient or odds ratio^a^	95% confidence interval	*p* value	Outcome	Coefficient or odds ratio^a^	95% confidence interval	*p* value
Appropriate parent involvement	1	**MTG: total**	− 0.48	− 0.76 to − 0.19	**0.001**	**MTG: total**	− 0.67	− 0.97 to − 0.37	**< 0.001**
	1	**MTG: environment**	− 0.57	− 0.92 to − 0.21	**0.001**	**MTG: environment**	− 0.71	− 1.17 to − 0.26	**0.006**
	1	**MTG: provider**	− 0.52	−0.82 to − 0.22	**0.001**	**MTG: provider**	− 0.75	− 0.99 to − 0.52	**< 0.001**
	1	**RTP: domestic**	[0.14^a^]	0.03 to 0.67	**0.01**	RTP: finances	[0.6^a^]	0.92 to 0.39	0.02
	1	**RTP: healthcare**	[0.35^a^]	0.17 to 0.72	**0.004**	WEMWBS	2.18	0.21 to 4.15	0.03
	1	RTP: services and aids	[0.42^a^]	0.18 to 0.97	0.04				
	2	**MTG: total**	− 0.60	− 1.00 to − 0.21	**0.003**				
	2	**MTG: environment**	− 0.65	− 1.08 to − 0.21	**0.004**				
	2	MTG: provider	− 0.48	− 0.90 to − 0.06	0.03				
	2	**MTG: process**	− 0.82	−1.32 to − 0.31	**0.002**				
	2	**WEMWBS**	4.47	1.96 to 6.97	**0.001**				
	3	**MTG: total**	− 0.87	0.45 to 1.29	**0.001**				
	3	**MTG: environment**	− 0.91	− 1.44 to − 0.37	**< 0.001**				
	3	**MTG: provider**	− 0.95	− 1.37 to − 0.52	**< 0.001**				
	3	MTG: process	− 0.63	− 1.18 to − 0.07	0.03				
	3	**WEMWBS**	3.45	0.92 to 5.99	**0.008**				
Promotion of health self-efficacy	1	**MTG: total**	− 0.51	− 0.87 to − 0.15	**0.006**	MTG: total	− 0.32	−0.62 to − 0.03	0.04
	1	**MTG: environment**	− 0.63	− 1.06 to − 0.18	**0.005**	MTG: environment	− 0.57	− 1.03 to − 0.11	0.02
	1	MTG: provider	− 0.47	− 0.85 to − 0.09	0.02	MTG: process	− 0.37	− 0.70 to − 0.04	0.03
	1	MTG: process	− 0.46	− 0.91 to − 0.01	0.04				
	1	RTP: finances	[0.26^a^]	0.07 to 0.93	0.04				
	1	**RTP: domestic**	[0.04^a^]	0.01 to 0.21	**0.001**				
	2	**MTG: total**	− 0.51	− 0.91 to − 0.11	**0.01**				
	2	MTG: provider	− 0.49	− 0.90 to − 0.08	0.02				
	2	**MTG: process**	− 0.70	− 1.21 to − 0.19	**0.007**				
	2	**MTG: total**	− 0.60	− 1.01 to − 0.20	**0.004**				
	3	**MTG: environment**	− 0.90	− 1.40 to − 0.40	**< 0.001**				
	3	**MTG: provider**	− 0.52	− 0.95 to − 0.11	**0.01**				
Meet adult team before transfer	1	**RTP: domestic**	6.29^a^	1.60 to 24.80	**0.009**	**RTP: education/employment**	2.33^a^	1.21 to 4.55	**0.01**
	1	**RTP: healthcare**	2.71^a^	1.24 to 5.90	**0.01**	RTP: finances	2.78^a^	1.10 to 7.14	0.03
	1	**RTP: services and aids**	5.15^a^	2.08 to 12.78	**< 0.001**	RTP: services and aids	2.50^a^	1.06 to 5.88	0.04
	1	RTP: transport	2.01^a^	1.06 to 3.79	0.03	Autonomy in appointments	1.60	0.32 to 2.87	0.02
	1	RTP: education/employment	3.24^a^	1.09 to 9.65	0.04				
	1	**Autonomy in appointments**	1.69	0.80 to 2.58	**< 0.001**				
	2	RTP: finances	2.64^a^	0.92 to 6.62	0.02				
	2	RTP: transport	2.08^a^	1.11 to 3.90	0.02				
	2	Autonomy in appointments	1.00	0.01 to 2.0	0.05				
	3	**RTP: healthcare**	2.11^a^	1.03 to 4.34	**0.004**				
	3	**Autonomy in appointments**	1.46	0.34 to 2.59	**0.01**				
Key worker	1	RTP: leisure	[0.56^a^]	0.32 to 0.96	0.04				
	2	**MTG: provider**	− 0.66	− 1.04 to − 0.28	**0.001**				
	2	**MTG: process**	− 0.69	− 1.17 to − 0.21	**0.005**				
	3	RTP: education/employment	[0.40^a^]	0.20 to 0.95	0.02				
Holistic life-skills training	1	RTP: domestic	[0.19^a^]	0.04 to 0.93	0.04				
	1	RTP: services and aids	[0.34^a^]	0.12 to 0.94	0.04				
	2	MTG: provider	[0.43]	0.03 to 0.84	0.04				
	3	MTG: total	− 0.46	− 0.87 to − 0.05	0.03				
	3	**MTG: provider**	− 0.57	− 0.99 to − 0.14	**0.009**				
	3	**RTP: domestic**	2.47^a^	1.10 to 5.58	**0.003**				
	3	RTP: romantic relationships	[0.52^a^]	0.26 to 0.98	0.04				
Written transition plan	1	RTP: romantic relationships	[0.43^a^]	0.19 to 0.96	0.04				
	2	MTG: total	− 0.72	− 1.39 to − 0.04	0.04				
	2	**MTG: process**	− 1.19	− 1.39 to − 0.04	**0.007**				
Coordinated team	1 3	RTP: domestic	[0.19^a^]	0.04 to 0.93	0.04	MTG: provider	− 0.67	− 1.25 to − 0.09	0.03
		RTP: healthcare	[0.17^a^]	0.04 to 0.82	0.03	RTP: education/employment	[0.31^a^]	0.11 to 0.82	0.02
	3	RTP: services and aids	[0.22^a^]	0.06 to 0.81	0.02	RTP: domestic	[0.41^a^]	0.19 to 0.91	0.03
Transition manager for clinical team		No associations				RTP: domestic	2.63^a^	1.16 to 5.88	0.02
						RTP: services and aids	[0.41^a^]	0.20 to 0.86	0.02
						RTP: romantic relationships	[0.52^a^]	0.31 to 0.89	0.02
Age-banded clinic	1	WEMWBS	3.08	0.18 to 5.98	0.04				
	1	**Autonomy in appointments**	1.44	0.48 to 2.4	**0.003**				
	2	RTP: education/employment	5.22^a^	1.21 to 22.53	0.03				

Note 1 Coefficients and odds ratios ‘year-by-year’ and for the consolidated PBF indicator have sub-optimal PBF delivery as the reference group

Note 2 A larger Mind the Gap score means less satisfaction with services than a smaller score

*MTG* Mind the Gap, *RTP* Rotterdam Transition Profile, *WEMWBS* Warwick Edinburgh Mental Wellbeing Scale

[] negative association, i.e. satisfactory PBF was associated with worse outcome

^a^ indicates an odds ratio

Bold indicates *p* ≤ 0.01

The pattern of associations was similar for ‘promotion of health self-efficacy’ though the evidence for the association was not as strong (total Mind the Gap *p* = 0.04) for the influence of the consolidated PBF indicator.

### PBFs as predictors of wellbeing scores

‘Appropriate parent involvement’ was associated with wellbeing (Warwick Edinburgh Mental Wellbeing Scale) in the second and third periods of the year-by-year analysis, though at a lesser level of significance (*p* = 0.03) for the consolidated PBF indicator.

### PBFs as predictors of the Rotterdam transition profile

‘Meeting the adult team before transfer’ was significantly associated (*p* ≤ 0.01) with a number of domains of the Rotterdam Profile in all three periods of the year-by-year analysis and also the consolidated PBF indicator (Table 3).

A number of weaker year-by-year and consolidated PBF indicator associations with other domains of the Rotterdam Profile were seen, but they were not in the predicted direction.

### PBFs as predictors of autonomy in appointments

There were significant associations (*p* < 0.01) of ‘meeting the adult team before transfer’ with ‘autonomy in appointments’ in periods 1 and 3 and with the consolidated PBF indicator (*p* = 0.02).

Thus, three PBFs of transitional healthcare had significant (*p* ≤ 0.01) positive associations with better outcomes, namely ‘appropriate parent involvement’, ‘promotion of health self-efficacy’ and ‘meeting the adult team before transfer’. The b-coefficients indicated changes of approximately 0.5 SDs with respect to the satisfaction with services scale (SD 1.5 in our population), wellbeing (SD 7.0 in our population) and autonomy in appointments (SD 3.0 in our population). The odds ratios indicated increased likelihoods of being in a more independent phase of transition. The other six PBFs had few statistically significant positive associations (*p* ≤ 0.01, Table 3) with better outcomes in the year-by-year analysis, had a number of negative associations, and had no positive associations with the consolidated indicator of exposure to PBFs.

## Discussion

Our study explored whether features of transitional healthcare, recommended in policy documents, were associated with positive outcomes. Three PBFs had significant positive associations with better outcomes, namely ‘appropriate parent involvement’, ‘promotion of health self-efficacy’, and ‘meeting the adult team before transfer’. The b-coefficients indicated clinically significant changes of approximately 0.5 SDs with respect to the satisfaction with services scale, wellbeing and autonomy in appointments. The odds ratios indicated increased likelihoods of being in a more independent phase of transition.

The other six PBFs had few statistically significant positive associations with better outcomes in the year-by-year analysis, had a number of negative associations and had no positive associations with the consolidated indicator of exposure to PBFs.

Two of the three key features which help (‘appropriate parent involvement’ and ‘promotion of health self-efficacy’) are not specific to transition; rather, they are features of developmentally appropriate healthcare for all young people. This finding reinforces the view that much of the essence of good transitional care is actually good developmentally appropriate healthcare [28, 29].

‘Appropriate parent involvement’ and ‘promotion of health self-efficacy’ were perceived to have been experienced satisfactorily by less than half of participants across transition across the three conditions. However, they were experienced by more young people with diabetes than by those with CP or ASD. For ‘meeting the adult team before transfer’, around two-thirds of young people with diabetes reported that they had met a member of the adult team but, for those with CP or ASD, it was less than a quarter. Thus, we found a different quality of experience of transitional healthcare for young people with a long-term illness (diabetes) compared to those with a long-term disability. These gaps in current practice need to be addressed through service development.

### Strengths and weaknesses

Our study was hypothesis driven, with pre-planned outcome measures that were applicable over condition and setting. These measures examined young people’s satisfaction with services, their wellbeing and participation, rather than focusing on process indicators such as attendance or loss to follow-up. There is a place for both outcomes and process indicators in transition evaluation. However, if service features do not improve the health or well-being of the young person, then it is hard to argue on clinical effectiveness grounds any basis for their adoption regardless of how process indicators may change. Our study has reported on wellbeing and participation which have rarely been used in this area, despite recommendations to do so [15, 17, 30]. Inclusion of a sample of young people with three contrasting conditions raises confidence in the generalisability of the findings to most young people with long-term health conditions. The retention rate of 73% to final visit was a considerable strength in this age group. Our data related to clinical practice for the three very different conditions, across several geographical locations. Unlike many previous studies [31–35], it was not an evaluation by a local team of their local intervention, which risks observer bias and limits generalisability of findings. Further, we collected data longitudinally over 3 years of transition. One limitation was lower average recruitment from a group with a special need for transitional care, namely those from areas of greater socioeconomic deprivation. However, the difference in overall IMD score on a continuous scale ranging from 0.5 to 87.8, was only 6.1; further, there was no difference in proportion of single parent families as compared to national norms. Regarding attrition, this was more marked by IMD score only for those with CP in Northern Ireland. There was, however, a reduction in the proportion of single parent families, which could be relevant to interpreting our finding that appropriate parent involvement in transition was significantly associated with better outcomes.

There was also lower than intended recruitment from the two disability groupings, so that analyses controlling for condition may have been underpowered. A further potential limitation was the accuracy of exposure to PBFs. The local researchers were trained together each year on this topic and then held the discussion each year with the young person, supplemented by the young person’s notes and inspection of medical notes. The analysis by whether exposure to the consolidated PBF indicator had been ‘optimal’ was a more demanding interrogation of the data than the year-by-year analyses because it required there to be some degree of good practice throughout the 3 years, not just over 1 year. However, the decision rules (described in Methods) about what constitutes optimal exposure to each PBF were determined by the research team and have some subjectivity.

### Strengths and weaknesses in relation to other studies

The three PBFs for which we found evidence of positive associations are supported by guidance and corroborating evidence discussed below. The link to current UK guidance (NICE) is also presented for each.

### ‘Appropriate parent involvement’

The NICE [4] guideline 43 emphasised ‘Appropriate parent involvement’ throughout its report; it is an overarching principle in section “Background”.1.1, and sections “Background”.2.19–1.2.22 focusing on the involvement of parents. Heath and Farre’s systematic review of studies of parents’ perceptions of their role in Transition [36] concluded that “*Parents can be key facilitators of their child’s healthcare Transition, supporting them to become experts in their own condition and care. However, to do so parents require clarification on their role and support from service providers*”. Akre’s study [37] found parental satisfaction with their involvement was associated with easier transition from the young person’s point of view. Allen’s study of young people with diabetes emphasised the importance of parents during Transition [38]. A review of qualitative studies [39] and a subsequent study [40] identified the tension that young people experience between seeking autonomy and still needing their parents. Two further recent reports investigated the parent–young person dyad [41, 42] and reached similar conclusions to those of the current study, namely that the parent and young person need to share care but that the change to adult autonomy is dynamic and will continuously change.

### ‘Promotion of health self-efficacy’

In section “Background”.2.17, NICE [4] recommended ‘Promotion of health self-efficacy’. Sattoe and van Staa [43] found that “*continuing attention to self-management*” was associated with better health-related quality of life. There is conflicting evidence as to whether a structured approach, including motivational techniques, to increase health self-efficacy in diabetes influences glycaemic control [44, 45]. After liver transplant, higher perceived self-management competence was actually associated with poorer clinical outcomes [46]. Mackie [47] showed the benefit of a 1-hour nurse-led intervention to promote knowledge and confidence about one’s condition, in this case congenital heart disease.

### ‘Meeting the adult team before transfer’

In sections “Background”.3.5 and 1.3.6, NICE [4] recommended ‘Meeting the adult team before transfer’. Our definition of meeting the adult team before transfer included clinics where adult and paediatric clinicians consulted jointly. In other studies, such joint clinics have shown improvements in certain indicators in nephrology (transplant rejection) [34], urology (aspects of care) [33] and rheumatology (knowledge of one’s condition) [48]. Crowley’s review [11] found joint clinics were associated with improved outcomes in those with diabetes.

The remaining six PBFs, for which our study found little evidence of benefit, had been included because a number of small published studies suggested they might be associated with improved outcomes. ‘Having a key worker’ was recommended by NICE [4] (sections “Background”.2.5–1.2.10 called for a ‘Named Worker’). Sloper et al. [49] found strong evidence for introducing key workers. The difficulty with ‘key worker’ may be operational rather than the principle; staff changes, due to leaving post, restrictions in job plans, service restructuring, sickness or maternity leave, make it difficult to provide a consistent key worker for all young people with long-term conditions. Having access to “*holistic life-skills training*” was recommended by NICE (sections “Background”.2.13–1.2.15). It was associated with greater satisfaction with service providers, and with more independent participation in domestic life in the third period of the year-by-year analysis, but not in the analysis by consolidated PBF indicators. Interpretation is difficult because few services provided this type of training. ‘Having a written transition plan’ was associated with greater satisfaction with services during the second study period but was otherwise negatively associated with many outcomes. NICE [4] (section “Background”.3.4) recommended transition planning but did not specifically mention that it should be a written document. Data from our qualitative work [50] showed the potential for careful planning to mitigate some of the disruptive, disorienting consequences of transfer. However, there were conflicting views about a ‘written’ plan. Some professionals said such plans get forgotten or lost, and personal interaction was far more important. On the other hand, lack of a formal plan left many families disoriented and wondering whether services would have sufficient resources to provide for care after transfer. Having a ‘transition manager for clinical team’ had no associations in the year-by-year analysis and had largely negative associations with the consolidated PBF indicator. In pilot studies, some benefits of having such a manager were seen after liver transplant [32] and in rheumatology [51].

Which outcomes should be measured in transition studies? [52–54] There is a need to go beyond current Delphi studies, and crucially involve young people and their families in developing consensus. Our choice of outcomes was informed by the International Classification of Functioning [18] and by discussion with international transition researchers, and conformed with many of the recommendations of subsequent international surveys [15, 16]. Although carefully chosen and piloted, measures may not be ideal; for example, we found the domain Finances of the Rotterdam Transition Profile not to be sensitive to change by the end of the study, as most young people remained dependent in part on family financial support.

## Conclusions

This study examined patient-level generic outcomes and provides new evidence about what may improve such outcomes. The findings are likely to be generalisable because participants had three very different conditions, attending services at many UK sites. Most previous studies have examined process indicators, which can be relevant to monitoring services but do not establish whether outcomes improve.

Three PBFs consistently associated with better outcomes were ‘Appropriate parent involvement’, ‘Promotion of health self-efficacy’ and ‘Meeting the adult team before transfer’. Our findings are relevant to almost all physicians and surgeons as some of their patients are likely to be adolescents and/or young adults with long-term conditions. The findings are also relevant for commissioners and managers of both child and adult health services who should prioritise changes for which there is evidence of benefit. There may need to be different approaches to different conditions as current provision of the three features is better for those with long-term conditions such as diabetes than for those with disabling conditions such as CP or ASD.

The three features should be introduced or maintained to a high standard in both child and adult services and the challenge of doing so evaluated. Such change will also require staff training and organisational change across child and adult healthcare services.

## Box 1 Definitions of Proposed Beneficial Features (PBFs)

**Age-banded clinic.** An intermediate clinic setting such as a young person’s clinic or a young adult team. In child health services, it would mean that children less than approximately 12 years would not be at the clinic. In adult services, it would mean adults over 24 years of age would not be at the clinic.

**Meet adult team before transfer.** This could be in a joint clinic where child and adult healthcare professionals consult together, or an adult clinician might visit the child clinic to be introduced, or the young person might have been taken to the adult clinic by their key worker or child healthcare professional to meet the adult clinician(s).

**Promotion of health self-efficacy.** The young person is asked ‘Have you received enough help to increase your confidence in managing your condition?’

**Written transition plan.** This should be created some time before transfer. It should include plans for wider aspects of transition, not just the arrangements for transfer to adult health services. The young person should have a copy of it and it should be reviewed at each appointment and updated as necessary.

**Appropriate parent involvement** in their child’s care, but with changing responsibilities. Parent and young person are asked separately if they think the level of involvement is appropriate. Involvement concerns what happens in the clinic (parent being present or not and who does the talking). It is the perceptions of both the young person and parent being satisfied with the level of parent involvement.

**Key worker.** This is a single person known to the young person whom they can easily contact or go to if there were any problems of co-ordination or misunderstandings that needed to be sorted out. The role could cross into education and social services. Whilst a clinic may have a policy to ‘appoint’ a key worker, this needs to be negotiated with the young person who may report it to be someone else they feel most comfortable with.

**Coordinated team.** Some young people need to see a team of people; for example, those with diabetes may need to see doctor, nurse, dietician and psychologist. Those with cerebral palsy may need to see doctor, physiotherapist and orthopaedic surgeon. The members of these teams need to work and communicate well together, and demonstrate to the young person and family that this is happening. Coordination of appointments on the same day is one demonstration of such coordination.

**Holistic life-skills training** about education, gaining employment, finances, housing, social relationships, sexual health, substance use, mental health, etc. as well as health maintenance. The young person is asked whether they have had any formal life-skill training offered relevant to their long-term condition. The health service may not provide such training but, during consultations, staff should inquire about such matters and make referrals to other agencies as needed.

**Transition manager for clinical team.** This person may not be known to the young person, but should facilitate good working relationships between adult and child services, ensure appropriate materials are available (such as for health education or the transition plan), and will monitor that the young person has a suitable appointment in adult services and whether the appointment is kept.

## Additional file

Additional file 1: **Table S1.** Demographic data for those at baseline and final visit. **Table S2.** Comparison of baseline and final visit scores for outcomes. (DOCX 50 kb)

## Abbreviations

ASD: autism spectrum disorder
CP: cerebral palsy
IMD: Index of Multiple Deprivation
NICE: National Institute for Health and Clinical Excellence
PBF: proposed beneficial feature
SD: standard deviation
