# Supplementary material for: A longitudinal, observational study of the features of transitional healthcare associated with better outcomes for young people with long-term conditions
Source: BMC Med. 2018 Jul 23;16:111. doi: 10.1186/s12916-018-1102-y (PMC6055340; doi:10.1186/s12916-018-1102-y)
Supplement: Supplementary file 1 — Table S1. Demographic data for those at baseline and final visit. Table S2. Comparison of baseline and final visit scores for outcomes. (DOCX 50 kb) [file 12916_2018_1102_MOESM1_ESM.docx]

**Additional file 1**

**A longitudinal, observational study of the features of transitional healthcare associated with better outcomes for young people with long-term conditions**

**Colver, A., Emeritus Professor of Community Child Health**

**McConachie, H., Emerita Professor of Child Clinical Psychology**

**Le Couteur, A., Professor of Child and Adolescent Psychiatry**

**Dovey-Pearce, G., Consultant Clinical Psychologist**

**Mann, K.D., Research Associate**

**McDonagh, J., Senior Lecturer in Paediatric and Adolescent Rheumatology**

**Pearce, M.S., Professor of Applied Epidemiology**

**Vale, L., Health Foundation Chair in Health Economics**

**Merrick, H., Research Assistant**

**Parr, J.R., Clinical Senior Lecturer in Paediatric Neurodisability**

**Table S1: Demographic data for those at baseline and final visit**

| **Young people** | | | | | **Baseline** | **Final visit** | **Loss to follow-up/drop outs** | **p-value** |
| --- | --- | --- | --- | --- | --- | --- | --- | --- |
|  | | | n | | 374 | 274 | 100 |  |
|  | | | % of original | | 100% | 73% | 27% |  |
| **Age** at first visit (years) | | | mean (SD) | | 16.2 (1.3) | 16.2 (1.3) | 16.2 (1.3) | 0.65^1^ |
|  |  |  | median (IQR) | | 16.3 (15.1-17.1) | 16.3 (15.1-17.1) | 16.1 (15.1-17.2) | 0.64^2^ |
| **Sex** | | Male | | n (%) | 219 (59%) | 158 (58%) | 61 (61%) | 0.56^3^ |
|  | | Female | |  | 155 (41%) | 116 (42%) | 39 (39%) |  |
| **Condition** | | D | | n (%) | 150 (40%) | 112 (41%) | 38 (38%) | 0.64^3^ |
|  | | CP | |  | 106 (28%) | 74 (27%) | 32 (32%) |  |
|  | | ASD | |  | 118 (32%) | 88 (32%) | 30 (30%) |  |
| **Recruitment site** | | | | | | | | |
| D | Site 1 | | | n (%) | 40 (27%) | 33 (29%) | 7 (7%) | 0.40^3^ |
| D | Site 2 | | |  | 17 (11%) | 10 (9%) | 7 (7%) |  |
| D | Site 3 | | |  | 40 (27%) | 31 (28%) | 9 (24%) |  |
| D | Site 4 | | |  | 30 (20%) | 22 (19%) | 8 (8%) |  |
| D | Site 5 | | |  | 23 (15%) | 16 (14%) | 7 (7%) |  |
| CP | Register 1 | | | n (%) | 49 (47%) | 39 (53%) | 10 (31%) | 0.06^3^ |
| CP | Register 2 | | |  | 46 (43%) | 30 (40%) | 16 (50%) |  |
| CP | Site 6 | | |  | 11 (10%) | 5 (7%) | 6 (19%) |  |
| ASD | Site 7 | | | n (%) | 40 (34%) | 31 (35%) | 9 (30%) | 0.6^3^ |
| ASD | Site 8 | | |  | 39 (33%) | 30 (34%) | 9 (30%) |  |
| ASD | Site 9 | | |  | 37 (31%) | 25 (28%) | 12 (40%) |  |
| ASD | Site 10 | | |  | 2 (2%) | 2 (2%) | 0 (0%) |  |
| **Overall socio-economic status determined by post code area** | | | |  | | | | |
| *England* | | | n (%) | | 328 (88%) | 244 (89%) | 84 (84%) |  |
| IMD score | | | median (IQR) | | 15.5 (8.6-28.0) | 15.3 (8.4-26.8) | 17.5 (8.9-29.9) | 0.31^2^ |
| *Northern Ireland* | | | n (%) | | 46 (12%) | 30 (11%) | 16 (16%) |  |
| MDM score | | | median (IQR) | | 15.3 (10.7-26.8) | 14.7 (9.6-18.1) | 26.6 (12.5-36.8) | 0.03^2^ |
| **Socio-economic factor** | | |  | | | | | |

| Type of parent | Birth mother/father | 364 (97.6%) | 268 (97.8) | 96 (96%) | 0.06^3^ |
| --- | --- | --- | --- | --- | --- |
|  | Foster parent | 9 (2.5%) | 1 (<1%) | 8 (8%) |  |
|  | Grand parent | 1 (<1%) | 0 (0%) | 1 (1%) |  |
|  | Step parent | 3 (<1%) | 0 (0%) | 3 (3%) |  |
|  | Did not take part | 7 (1.3%) | 5 (1.8%) | 2 (2%) |  |
| Marital status | Married | 280 (75%) | 218 (79%) | 62 (62%) | 0.004^3^ |
|  | Divorced | 56 (15%) | 35 (13%) | 21 (21%) |  |
|  | Single | 25 (6.7%) | 13 (4.7%) | 12 (12%) |  |
|  | Widowed | 6 (1.6%) | 3 (1.1%) | 3 (3%) |  |
|  | Did not take part | 7 (1.9% | 5 (1.8%) | 2 (2%) |  |

^1^t-test between final visit sample and drop outs

^2^Mann-Whitney test between final visit sample and drop outs

^3^Chi-squared test between final visit sample and drop outs

SD: Standard Deviation; IQR: Inter-quartile range; D: diabetes; CP: cerebral palsy; ASD: autism spectrum disorder

IMD: Index of Multiple Deprivation; MDM: Multiple Deprivation Measure

**Table S2: Comparison of baseline and final visit scores for outcomes**

|  |  |  | **Baseline** | | **Final visit** | |  |  |
| --- | --- | --- | --- | --- | --- | --- | --- | --- |
| **Outcome** | **Condition** | **n** | **Median** | **IQR** | **Median** | **IQR** |  | **p value*** |
| **Satisfaction with services.**  **Mind the Gap**  **Young person report**  (For Mind the Gap and its subscales, higher scores indicate less satisfaction) |  |  |  |  |  |  |  |  |
|  | All | 235 | 0.57 | 0.00, 1.38 | 1.00 | 0.29, 2.00 |  | <0.001 |
| Overall satisfaction with services | D | 109 | 0.57 | 0.00, 1.19 | 0.67 | 0.05, 1.30 |  | 0.13 |
|  | CP | 54 | 0.63 | -0.05, 2.00 | 1.41 | 0.38, 2.19 |  | 0.04 |
|  | ASD | 72 | 0.69 | 0.05, 1.60 | 1.78 | 0.87, 2.53 |  | <0.001 |
|  | All | 235 | 0.80 | 0.00, 1.80 | 1.00 | 0.20, 2.10 |  | <0.001 |
| Management of the environment | D | 109 | 0.80 | 0.00, 1.60 | 0.80 | 0.00, 1.60 |  | 0.36 |
|  | CP | 54 | 0.60 | -0.40, 2.20 | 1.80 | 0.40, 2.80 |  | 0.02 |
|  | ASD | 72 | 0.80 | 0.00, 1.80 | 1.60 | 0.80, 2.80 |  | <0.001 |
|  | All | 235 | 0.36 | -0.09, 1.18 | 0.81 | 0.00, 1.81 |  | <0.001 |
| Provider characteristics | D | 109 | 0.36 | -0.18, 1.09 | 0.36 | -0.09, 1.18 |  | 0.12 |
|  | CP | 54 | 0.33 | 0.00, 1.27 | 0.73 | 0.00, 2.09 |  | 0.15 |
|  | ASD | 72 | 0.64 | 0.00, 1.55 | 1.55 | 0.55, 2.40 |  | <0.001 |
|  | All | 235 | 0.60 | 0.00, 1.80 | 1.25 | 0.40, 2.50 |  | <0.001 |
| Process Issues | D | 109 | 0.60 | 0.00, 1.40 | 0.80 | 0.20, 1.60 |  | 0.20 |
|  | CP | 54 | 0.80 | -0.40, 3.20 | 2.00 | 0.50, 3.40 |  | 0.11 |
|  | ASD | 72 | 1.00 | 0.10, 2.10 | 2.33 | 1.00, 3.22 |  | <0.001 |
| **Mental well-being.**  **WEMWBS** | All | 271 | 51.00 | 45.00, 56.00 | 51.00 | 45.00, 56.00 |  | 0.43 |
| Total score | D | 111 | 53.00 | 49.00, 58.00 | 54.00 | 46.00, 57.00 |  | 0.97 |
|  | CP | 74 | 53.00 | 49.00, 58.00 | 52.00 | 47.00, 56.00 |  | 0.71 |
|  | ASD | 86 | 46.00 | 39.00, 52.00 | 47.00 | 42.00, 54.00 |  | 0.12 |
| **Autonomy in appointments.** | |  | *** |  |  |  |  |  |
|  | All | 262 | 8.00 | 5.50, 0.00 | 10.00 | 6.00, 13.00 |  | <0.001 |
| Total score | D | 109 | 9.00 | 7.00, 1.00 | 12.00 | 9.00, 14.00 |  | <0.001 |
|  | CP | 69 | 7.00 | 5.00, 9.00 | 9.00 | 6.00, 12.00 |  | <0.001 |
|  | ASD | 84 | 6.00 | 5.00, 8.00 | 7.00 | 5.00, 11.00 |  | 0.01 |
| **Participation.**  **Rotterdam Transition Profile** | |  | **n (%) of young people in phase 3** | | **n (%) of young people in phase 3** | |  | **p value**** |
|  | All | 273 | 4 (2) | | 45 (16) | |  | <0.001 |
|  | D | 112 | 3 (3) | | 29 (26) | |  | <0.001 |
| Education/employment | CP | 74 | 0 (0) | | 7 (9) | |  | 0.008 |
|  | ASD | 87 | 1 (1) | | 9 (10) | |  | 0.01 |
|  | All | 273 | 14 (5) | | 61 (22) | |  | <0.001 |
|  | D | 112 | 4 (4) | | 30 (27) | |  | <0.001 |
| Finances | CP | 74 | 8 (11) | | 12 (16) | |  | 0.21 |
|  | ASD | 87 | 2 (2) | | 19 (22) | |  | <0.001 |
|  | All | 274 | 0 (0) | | 39 (14) | |  | <0.001 |
|  | D | 112 | 0 (0) | | 24 (22) | |  | <0.001 |
| Domestic | CP | 74 | 0 (0) | | 4 (5) | |  | 0.05 |
|  | ASD | 88 | 0 (0) | | 11 (13) | |  | 0.001 |
|  | All | 270 | 63 (24) | | 130 (49) | |  | <0.001 |
|  | D | 112 | 31 (28) | | 62 (57) | |  | <0.001 |
| Romantic relationships | CP | 73 | 8 (11) | | 26 (37) | |  | <0.001 |
|  | ASD | 85 | 24 (28) | | 49 (41) | |  | <0.001 |
|  | All | 274 | 55 (20) | | 157 (57) | |  | <0.001 |
|  | D | 112 | 26 (23) | | 86 (77) | |  | <0.001 |
| Transport | CP | 74 | 9 (12) | | 30 (41) | |  | <0.001 |
|  | ASD | 88 | 20 (23) | | 41 (47) | |  | <0.001 |
|  | All | 271 | 125 (46) | | 166 (61) | |  | <0.001 |
|  | D | 112 | 66 (59) | | 89 (79) | |  | <0.001 |
| Leisure | CP | 74 | 37 (50) | | 42 (57) | |  | 0.02 |
|  | ASD | 85 | 22 (26) | | 35 (41) | |  | 0.007 |
|  | All | 274 | 14 (5) | | 66 (24) | |  | <0.001 |
|  | D | 112 | 9 (8) | | 51 (46) | |  | <0.001 |
| Healthcare | CP | 74 | 2 (3) | | 5 (7) | |  | 0.18 |
|  | ASD | 88 | 3 (3) | | 10 (11) | |  | 0.05 |
|  | All | 261 | 3 (1) | | 33 (14) | |  | <0.001 |
|  | D | 112 | 2 (2) | | 22 (27) | |  | <0.001 |
| Services and aids | CP | 70 | 0 (0) | | 2 (3) | |  | 0.16 |
|  | ASD | 79 | 1 (1) | | 9 (11) | |  | 0.005 |

*p value from Wilcoxon matched pairs signed rank test

**p value from McNemar’s chi-squared test

NB p-values for the total sample do not account for the different conditions.

***These values are from visit 2 (not baseline) as the instrument was not introduced until visit 2 (see Methods)

IQR: Inter-quartile Range; D: diabetes; CP: cerebral palsy; ASD: autism spectrum disorder

phase 3 = independent functioning

WEMWBS Warwick Edinburgh Mental Wellbeing Scale
